# The attitudes and beliefs of Pakistani medical practitioners about depression: a cross-sectional study in Lahore using the Revised Depression Attitude Questionnaire (R-DAQ)

**DOI:** 10.1186/s12888-016-1069-1

**Published:** 2016-10-18

**Authors:** Mark Haddad, Ahmed Waqas, Wahhaj Qayyum, Maryam Shams, Saad Malik

**Affiliations:** 1 Centre for Mental Health Research, School of Health Sciences, City University London, Northampton Square, London, EC1V 0HB UK; 2 East London NHS Foundation Trust, London, UK; 3 CMH Lahore Medical College and Institute of Dentistry, Lahore, Pakistan

## Abstract

**Background:**

Mental disorders such as depression are common and rank as major contributors to the global burden of disease. Condition recognition and subsequent management of depression is variable and influenced by the attitudes and beliefs of clinicians as well as those of patients. Most studies examining health professionals’ attitudes have been conducted in Western nations; this study explores beliefs and attitudes about depression among doctors working in Lahore, Pakistan.

**Methods:**

A cross-sectional survey conducted in 2015 used a questionnaire concerning demographics, education in psychiatry, beliefs about depression causes, and attitudes about depression using the Revised Depression Attitude Questionnaire (R-DAQ). A convenience sample of 700 non-psychiatrist medical practitioners based in six hospitals in Lahore was approached to participate in the survey.

**Results:**

Six hundred and one (86 %) of the doctors approached consented to participate; almost all respondents (99 %) endorsed one of various biopsychosocial causes of depression (38 to 79 % for particular causes), and 37 % (between 13 and 19 % for particular causes) noted that supernatural forces could be responsible. Supernatural causes were more commonly held by female doctors, those working in rural settings, and those with greater psychiatry specialist education. Attitudes to depression were mostly less confident or optimistic and less inclined to a generalist perspective than those of clinicians in the UK or European nations, and deterministic perspectives that depression is a natural part of aging or due to personal failings were particularly common. However, there was substantial confidence in the efficacy of antidepressants and psychological therapy. More confident and therapeutically optimistic views and a more generalist perspective about depression management were associated with a rejection of supernatural explanations of the origin of depression.

**Conclusions:**

Non-psychiatrist medical practitioners in Pakistan hold a range of views about the causes of depression, with supernatural explanations held by more than a third. Depression attitudes appear less positive than among UK and European clinicians, with the notions that depression is due to a lack of stamina and will-power and a natural part of growing old being especially commonly held; more positive attitudes appear to be associated with a rejection of supernatural explanatory models of depression.

## Background

Mental disorders occur commonly throughout the world, and are a major and often underappreciated cause of global morbidity and mortality. In the most recent World Health Organisation burden of disease study (GBD, 2010), mental, neurological and substance use disorders accounted for one-tenth (10.4 %) of global disability-adjusted life years (DALYs), and 28.5 % of global years lived with disability (YLDs), making them the leading cause of YLDs. Mental and substance use disorders make up the largest proportion of this (71.4 % of DALYs) [1].

Common mental disorders, in particular depression, account for the bulk of the disability burden attributable to mental illness in both developed and developing countries; this relates to the high prevalence and risks of recurrence and persistence of depression coupled with its pervasive effect on function. Major depressive disorder currently ranks as the second leading cause of global disease burden by YLD; it is the leading cause in 56 countries, the second leading cause in 56 countries, and the third in 34 countries [2].

Mental disorders exist in all countries and, together with the availability of healthcare structures and resources, the beliefs and attitudes of public and professionals are an important determinant of health outcomes, strongly influencing the way people perceive and experience their mental health problems and the accessibility and quality of the health care they receive [3]. Mental health related stigma is apparent in both developed and developing nations: widely-held misconceptions about mental illness among the public result in prejudice and discrimination, whilst for those with mental illness they reduce hope and esteem [4], act as a deterrent to mental illness disclosure and inhibit help-seeking [5]. Negative attitudes commonly held by the general public concern the difference, unpredictability, and dangerousness of people with mental illness, together with a disinclination for social closeness and a desire for measures to restrict liberty. Research indicates that these stigmatising attitudes are held, albeit to varying degrees, by health professionals as well as the public [6], and are evident in Western and non-Western societies [7].

Culture influences the concept and representations of illness, affecting the presentation, assessment and management of mental disorders. The definition and classification of mental disorders is predominantly Western-based and this can conflict with cultural contexts where alternative conceptions and traditional explanation of these health problems may be commonly held [1]. Supernatural attributions for the cause and treatment of mental illness, whilst relatively uncommon among Western cultures, are a more frequently held explanatory model among other ethnic groups. Studies conducted with mental health service users in the United Kingdom (UK) from different ethnic and cultural backgrounds show that mystical, religious and magical explanations for mental illness are substantially more likely to be cited by those who are Asian or West African, whereas White patients more commonly attribute mental illness to biological factors [8]. Studies indicate that supernatural explanatory models appear to be associated with reduced treatment satisfaction and poorer therapeutic relationships with health professionals [8] as well as a greater likelihood to seek help from faith-based organisations [9]. This accords with systematic review evidence derived from population surveys in Australia, the USA and European nations that biological and genetic conceptualisations of mental illness are associated with greater endorsement of treatment by medical professionals [10]. However, public beliefs about the nature and cause of mental illness influence social acceptance, and although biological explanations may facilitate better satisfaction with conventional treatments and health services, they also appear to be associated with a greater desire for social distance from people with mental health problems [10, 11].

The notion sometimes voiced that mental health related stigma is less evident in Asian and African nations [12] or that it rarely occurs in Islamic societies [13] is not supported by the available research findings, which consistently show that negative views are prevalent in these cultures. Indeed, some stigmatizing views about those with mental illness such as perceived dangerousness and the desire for social distance appear more strongly held than in the responses to similar surveys in Western societies. It is likely that these views are shaped by complex and interlinked factors such as cultural understanding, education including mental health literacy, personal contact and experience of mental health problems, and the availability and provision of mental health treatment [7, 14].

Culture informs health professionals’ understanding of mental illness as well as the views of the lay public, with studies indicating that these attitudes within a particular culture are broadly similar irrespective of occupation and role [15]. There are fewer studies of health professionals’ attitudes or opinions towards individuals with mental disorders than those of the general public: Schulze [6] notes 65 such general population surveys conducted up to 2006 compared with only 9 surveys of mental health professionals. A more recent review [3] identified 25 studies examining whether mental health professionals held stigmatizing views towards people with mental disorders. Most of the studies of health professionals have been conducted with in Western nations, and these have frequently involved medical students. There are fewer studies of the views of health professionals from non-Western populations, though these have been conducted in Nigeria [16], Sri Lanka [17], Malaysia [18], Bangladesh [19], and Pakistan [20].

Comparisons of attitudes between health professionals and the general public in Western settings indicate broad similarities between groups, though there is some conflicting evidence: a large-scale US study identified more positive views among professionals despite the persistence of stereotypes [21], whereas a similarly sized Australian survey found more pessimistic views about mental illness among psychiatrists and general practitioners than the public [22]. In general, measures of social distance show little differences between health professionals and the public, however professionals typically show less socially restrictive attitudes [3]. Likewise, studies in non-Western countries indicate general similarities between the views of health professionals and comparator population groups such as university students or the general public [7, 23, 24].

Research concerning the views of health professionals in non-Western nations is limited, and to date few studies have considered the relationship between causal explanations for mental disorders and attitudes to condition treatment among clinicians.

This study aimed to explore the attitudes and beliefs of a sample of medical practitioners in Pakistan about the causes of depression and their attitudes to its management. We examined the relationship between (1) culturally informed explanatory models of depression, (2) depression attitudes, comprising: therapeutic optimism about depression, professional confidence in its management, and the degree to which a generalist perspective about its occurrence, recognition and treatment is held, and (3) demographic and training characteristics of participants (including gender, specialism, practice setting, psychiatric training and experience).

## Methods

This was a cross-sectional study using a self-complete survey questionnaire distributed to a convenience sample of medical practitioners based at six hospitals in Lahore, Pakistan.

### Setting

Pakistan is a South Asian nation with population of 185.1 million; it is classed as a low income nation based on World Bank 2004 criteria, and the Human Development Index (HDI) (a summary measure for assessing healthy life expectancy, access to knowledge and standard of living) ranking is 147 out of 188 countries and territories, with high levels of gender inequality (ranked 121 out of 155 countries); life expectancy from birth is 66.2 [25]. Total expenditure on health is around 2.2 % of GDP [26], and only 0.4 % of health expenditure is devoted to mental health [27].

Lahore is the capital city of the province of Punjab, and the second largest city in Pakistan, with an estimated population of 7.6 million. This survey was administered at six hospitals in Lahore: Mayo Hospital, Jinnah Hospital, Combined Military Hospital and Services Hospital, Doctors Hospital, and Lahore General Hospital, between June 2015 and September 2015.

### Measures

A single self-report questionnaire consisting of three sections was used. The sections concerned:

1. Participant demographics and medical specialism, including involvement in specialist psychiatry training and frequency of encountering depression in their practice;
2. The possible causes of depression, with a format previously used in a study of university students’ stigma-related views about mental illness [28], in which participants selected from among 13 responses relating to biological, psycho-social, and religious/supernatural factors, with multiple responses allowed;
3. The Revised Depression Attitude Questionnaire (R-DAQ) [29], a 22-item scale designed to measure clinicians’ attitudes to depression, and derived from the depression attitude questionnaire’ (DAQ) which was developed in the UK [30] for General Practitioners (GPs). The R-DAQ comprises 22 attitude statements with response options noting level of agreement scored with a five-point Likert scale. It has previously been used with a sample of 1193 health professionals (almost entirely from the UK), with psychometric adequacy evident: total scale internal consistency 0.84, clear construct validity, easy readability, and minimal floor and ceiling effects. Three sub-scales were evident in the R-DAQ development sample concerning respondents’ attitudes about “professional confidence in depression care”, “therapeutic optimism/pessimism about depression” and “generalist perspective about depression occurrence, recognition and management”.

The survey questionnaire was pilot tested with six Pakistani medical students to ensure that the phrasing, terminology, layout, and time taken to complete the survey were clear, understandable and appropriate to the target population of Pakistani medical practitioners. Only minor modifications of the demographic items were required: the depression causes section had previously been used with a similar sample; and there were no issues with the readability of the R-DAQ (previous examination established the Flesch-Kincaid Grade level was 9.4, indicating it to be understandable to a typical 14–15 year-old student).

### Procedure

Ethical approval for the study was sought and provided by the Ethical Review Committee of CMH Lahore Medical College, Lahore, Pakistan. Convenience sampling was employed and all available medical practitioners, excluding any who had formal diploma or fellowship training in psychiatry or were practicing as psychiatrists, were approached by four medical students who visited the sites on repeated occasions over a three month period. These researchers provided study information, consent form and the survey questionnaire; potential participants were ensured anonymity and informed that only group findings would be reported.

#### Analysis

The sample size was determined on the basis of several calculations. In relation to the dichotomous questionnaire items used in this survey, with application of the most conservative estimate of response distribution, a sample of 383 was required to provide 5 % precision and a 95 % confidence interval for differences within the sample. A calculation based on the R-DAQ development study mean (overall scale) and standard deviation values for GPs compared to other health professionals (counsellors and psychologists) [29], indicated that a total sample of 326 was sufficient at 90 % power for 5 % significance level (two-sided test). Finally, values derived from the difference between GPs according to age group using the original DAQ overall score [31] provided a sample estimation of 412 (again, based on 90 % power and 5 % significance), whilst using single items of the original DAQ retained in the R-DAQ, indicated samples sizes of 134 (item 11) and 176 (item 15). To account for probable recruitment we considered the response rates in similar studies conducted within a similar population [20, 28], and anticipated that a total of 700 medical practitioners would need to be contacted to provide our required sample.

All data were analysed in SPSS v. 22 [32]. Descriptive statistics of the demographic, attitude and casual belief variables were indicated with mean, standard deviation (SD), number (N) and percentage (%) as appropriate. The factor structure of the R-DAQ in this population was examined using Principle Axis Factoring (PAF) and oblique rotation using the Direct Oblimin method, and also with the FACTOR programme [33] which enabled analyses based on a polychoric correlation matrix. The adequacy of factor solutions was assessed on the basis of Eigen values, percentage of variance explained, theoretical coherence of factors, simplicity of the factor loadings and Catell’s scree plot; additionally we considered solutions in light of the factor structure evident in the (largely UK) R-DAQ initial sample. Summary details of the psychometric properties of the R-DAQ are provided in this paper, and a more detailed description of this analysis is reported elsewhere [34]. Chi-square tests were used to examine differences in relation to respondent characteristics and beliefs about the causes of depression. Independent sample t-tests were used to examine the association of R-DAQ scores with demographic and training variables. Multiple linear regression analysis was used to identify associations between demographic variables, views about the depression causes, and the R-DAQ subscales. All the statistical tests were two-sided and the significance was set at *p* < 0.05. For the regression analysis, multicollinearity was examined by obtaining the variance inflation factor (VIF) and tolerance values for all the variables after running the regression, with a tolerance of less than 0.10 and a VIF of 5 or 10 and above indicating a problem [35].

## Results

### Respondent characteristics: demographics, training and practice

A total of 601 (85.9 %) of the medical practitioners contacted returned the questionnaire, and the frequency of missing values was between 1 and 1.5 % for quantitative variables and 0 and 14 % for categorical variables (supernatural causes for depression were the most commonly omitted items). The mean age was of survey participants was 29.7 (SD = 7.8, range 22 to 61), with male respondents slightly older (30.5) than females (29.0). Just over half (52 %) had graduated since 2011. As may be seen in Table 1, slightly more (52 %) of the participants were female, and around a third (36 %) had studied psychiatry as a major subject in medical school. More than two-thirds (69 %) planned to attend continuing medical education (CME) courses in psychiatry in the future (moderate or high priority), and nearly half (49 %) noted that they relatively frequently encountered depression in their practice.

**Table 1:** Characteristics of survey participants (*n* = 601)

Variables		Frequency (%)
Gender	Male	286 (47.6)
	Female	315 (52.4)
Speciality	Medicine/Paeds	394 (65.6)
	Surgery/ObsGyn	207 (34.4)
Last Degree	MBBS	474 (78.9)
	Postgraduate	125 (20.8)
Practice setting	Rural	78 (13.0)
	Urban	523 (87.0)
Have you studied psychiatry as a major subject in medical school? (Yes/No)	Yes	217 (36.1)
Have you had an internship experience in Psychiatry? (Yes/No)	Yes	121 (20.1)
Have you ever taken CME courses? (Yes/No)	Yes	298 (49.6)
Have you ever taken CME courses in Psychiatry? (Yes/No)	Yes	143 (23.8)
Have you ever read an article on Psychiatry? (Yes/No)	Yes	202 (33.6)
Have you studied abroad? (Yes/No)	Yes	102 (17.0)
Do you plan to attend CME courses in in future?	Low priority	99 (16.5)
	Moderate priority	232 (38.6)
	High priority	262 (43.6)
Do you plan to attend CME courses in Psychiatry in future?	Low priority	180 (30.0)
	Moderate priority	243 (40.4)
	High priority	172 (28.6)
How frequently do you encounter depression in your practice setting?	Never	31 (5.2)
	Rarely	74 (12.5)
	Occasionally	198 (33.4)
	Moderately	153 (25.8)
	Quite often	137 (23.1)

### Respondent characteristics and beliefs about the causes of depression

Participant responses to questionnaire items concerning their agreement or disagreement about a range of causes of depression showed that biopsychosocial explanations were most commonly held, with stresses related to work, study, divorce, and poverty regarded by two-thirds or more of respondent as depressogenic. As may be seen in Table 2, almost all (98.5 %) of the respondents endorsed one or more of the biopsychosocial causes; experiencing work-related stress and traumatic events were the items that attracted most agreement.

**Table 2:** Beliefs concerning the causes of depression in ranked order (*n* = 601)

		No. in agreement (%)
Biopsychosocial causes	Work related stress	462/584 (79.1)
	Traumatic events	409/563 (72.6)
	Poverty	400/567 (70.5)
	Studies related stress	398/579 (68.7)
	Divorce	380/564 (67.4)
	Physical abuse	274/538 (50.9)
	Misuse of drugs	244/533 (45.8)
	Genetic	214/538 (39.8)
	Misuse of alcohol	199/525 (37.9)
	Any biopsychosocial cause	591/600 (98.5)
Religious and supernatural causes	Evil eye	99/516 (19.2)
	God’s punishment	94/520 (18.1)
	Black magic	69/514 (13.4)
	Jinn possession	69/517 (13.3)
	Any religious or supernatural cause	198/532 (37.2)

Although less commonly held, over a third of the sample (37.2 %) indicated one or more supernatural cause for the development of depression, with the evil eye (19.2 %) and punishment from God (18.1 %) most frequently endorsed.

Belief in any supernatural cause (compared with none) was not associated with medical speciality, but was much more common among doctors whose clinical practice was in a rural rather than urban setting (51.9 v 34.7 %) *X* ^2^ = 8.360, *p* = 0.004, and those of female gender (41.7 v 32.4 %) *X* ^2^ = 4.858, *p* = 0.028. Somewhat surprisingly, belief in any supernatural cause was more common among doctors who had studied psychiatry as a major subject (42.9 v 33.6 %) *X* ^2^ = 4.652, *p* = 0.031, undertaken continuing medical education in psychiatry (48.6 v 33.2 %) *X* ^2^ = 10.242, *p* = 0.001, and read an article on psychiatry (47.1 v 31.4 %) *X* ^2^ = 12.995, *p* < 0.001. Neither respondent age nor the year of graduation appeared associated with likelihood of stating particular causal beliefs. When all the relevant respondent variables were controlled in a multiple regression analysis, only rural practice and having read a psychiatry article remained significant predictors of holding beliefs in supernatural causes for depression.

### Attitudes to depression

Respondents’ attitudes to depression were measured with the R-DAQ. The characteristics of this measure were examined within this group by exploratory factor analysis which is reported in detail in a further publication [34]. As in the initial R-DAQ study [29], three factors were evident, however several items fitted poorly within the total scale and sub-scales and the optimal final solution for this population contained 15 items (5 items in each of the subscales) which explained (using FACTOR based on polychoric correlation, Unweighted Least Squares with Promin rotation) 46.3 % of the scale variance. The scale internal consistency was found to be lower for this population than in the predominantly UK R-DAQ development sample: for the total scale, standardised Cronbach’s alpha was 0.69; the subscale reliability estimates [36] were, for the generalist perspective sub-scale 0.77, for the professional confidence sub-scale 0.73, and for the therapeutic optimism/pessimism sub-scale 0.64.

As may be seen in Table 3, attitudes to depression measured by the R-DAQ indicated a rather pessimistic and deterministic view, with respondents generally neutral or in agreement with items measuring the extent of therapeutic optimism (the negatively phrased items are reverse scored when the overall R-DAQ scale is used, but are presented here without this change). Most survey participants considered depression to be related to a lack of will-power (67.2 %) or to poor stamina (56.8 %), and nearly half (42.9 %) agreed that it was a natural part of growing old. These negative views about the nature of depression contrasted with responses to items about depression management: very few respondents indicated agreement with the notion that antidepressant (8.6 %) or psychological therapies (13 %) were ineffective treatments, and less than one-fifth felt that depression was unamenable to change or that there was little to be offered if initial treatments did not succeed.

**Table 3:** Responses to the R-DAQ items (with no item reverse scoring) ranked by extent of agreement (agreement = combining *agree* and *strongly agree*)

Depression attitudes: R-DAQ factors and items	No in agreement	Percent	Mean	Std. deviation
Professional confidence in depression care				
19: It is rewarding to spend time looking after depressed patients	356/599	59.4	3.50	0.98
8: I am more comfortable working with physical illness than with mental illnesses like depression (R)	356/601	59.2	3.52	1.09
11: My profession is well placed to assist patients with depression	328/599	54.8	3.37	1.07
7: I feel confident in assessing depression in patients	323/598	54.0	3.32	0.99
1: I feel comfortable in dealing with depressed patients’ needs	320/601	53.2	3.29	1.03
15: My profession is well trained to assist patients with depression	265/600	44.2	3.13	1.08
17: I feel confident in assessing suicide risk in patients presenting with depression	220/599	36.7	3.00	1.08
Therapeutic optimism/pessimism about depression				
5: One of the main causes of depression is a lack of self-discipline and will-power (R)	402/598	67.2	3.65	1.06
12: Becoming depressed is a way that people with poor stamina deal with life difficulties (R)	339/597	56.8	3.38	1.07
9: Becoming depressed is a natural part of being old (R)	257/599	42.9	3.02	1.17
13: Once a person has made up their mind about taking their own life no one can stop them (R)	182/599	30.4	2.56	1.23
6: Depression treatments medicalise unhappiness (R)	168/592	28.4	2.97	0.92
20: Becoming depressed is a natural part of adolescence (R)	145/600	24.2	2.58	1.06
18: Depression reflects a response which is not amenable to change (R)	115/597	19.3	2.48	0.99
21: There is little to be offered to depressed patients who do not respond to initial treatments (R)	111/598	18.6	2.47	1.04
3: Psychological therapy tends to be unsuccessful with people who are depressed (R)	78/601	13.0	2.15	0.96
4: Antidepressant therapy tends to be unsuccessful with people who are depressed (R)	51/596	8.6	2.06	0.88
Generalist perspective about depression occurrence, recognition and management				
22: Anyone can suffer from depression	500/599	83.5	4.06	1.03
10: All health professionals should have skills in recognising and managing depression	478/600	79.7	3.99	0.99
16: Recognising and managing depression is often an important part of managing other health problems	463/600	77.2	3.86	0.98
14: People with depression have care needs similar to other medical conditions like diabetes, COPD or arthritis.	417/599	69.6	3.64	1.15
2: Depression is a disease like any other (e.g. asthma, diabetes)	359/601	59.7	3.38	1.39

*R* indicates items that are reversed for summary scale and sub-scale scoring

Views about a generalist approach to depression and its management attracted general agreement, and the notion that anyone could suffer from depression was particularly strongly endorsed, attracting agreement from 83.5 % of the sample. Professional confidence in depression management was limited: most respondents (59.2 %) indicated that they were more comfortable working with physical rather than mental health problems, and only around a half or less noted confidence about their abilities or training to assess and manage depression.

### Relationship between depression attitudes and respondent characteristics

No significant associations were evident between gender, medical specialism, having studied abroad, or frequency of clinical work with people with depression, and attitude scores measured by R-DAQ sub-scales. However, those respondents who identified themselves as working predominantly in a rural rather than urban setting had less of a generalist perspective (*t* = 3.97, d.f. = 87, *p* < 0.001) and less optimism about the response of depression to treatment (*t* = 2.95, d.f. = 568, *p* = 0.003). Respondents who had studied psychiatry as a major topic or undertaken psychiatry CME were significantly less likely to hold a generalist perspective about depression (*t* = 5.49, d.f. = 332, *p* < 0.001); and greater professional confidence in delivering depression care was also evident among clinicians who had undertaken psychiatry CME (*t* = 2.41, d.f. = 593, *p* = 0.016), as well as among those who had graduated since 2012 (*t* = 3.81, d.f. = 590, *p* < 0.001).

### Relationship between depression attitudes and causal beliefs

Examination of the relationship between R-DAQ (total and sub-scale) scores and explanatory beliefs for depression indicated particular associations.

As may be seen in Table 4, a significantly higher score on the total R-DAQ scale and on each of the three sub-scales – indicating a more positive and inclusive view of depression and its management - was evident among those participants who eschewed all supernatural beliefs about depression causation (compared to those who endorsed one or more supernatural explanation). The extent of difference due to holding supernatural beliefs about depression was greatest for the generalist perspective sub-scale (*M* = 1.74, SD = 0.362).

**Table 4:** Association between depression attitudes (R-DAQ) and supernatural causal beliefs

	Any supernatural causal belief (*n* = 333)	No supernatural causal belief (*n* = 197)	Independent samples *t*-test	
Depression attitudes (R-DAQ)	Mean (SD)	Mean (SD)	*t*	Sig. (2-tailed)
Professional confidence (R-DAQ items 1,7,11,15,17)	15.71 (3.22)	16.34 (3.55)	2.045	0.041
Therapeutic optimism (R-DAQ items 3,4,18,20,21)	17.65 (2.73)	18.61 (2.89)	3.748	<0.001
Generalist perspective (R-DAQ items 2,10,14,16,22)	17.75 (4.50)	19.49 (3.09)	4.796	<0.001
Total R-DAQ (15 items)	51.21 (6.61)	54.51 (6.08)	5.758	<0.001
Total R-DAQ (22 items)	71.33 (6.43)	74.96 (7.36)	5.584	<0.001

Linear regression was used to further explore the relationships between respondent characteristics, and their causal beliefs and attitudes to depression. Using the R-DAQ overall score as dependent variable and entering predictor variables based on the preceding analyses, provided a model which explained 10 % of the variance in the R-DAQ score (R^2^ = 0.095), F_3,513_ = 17.907, *p* < 0.001. Rejecting supernatural causal beliefs accounted for the largest positive effect on R-DAQ score (β = 0.247), and practice in urban rather than rural settings and noting divorce as a cause were included in the final model (Table 5).

**Table 5:** Linear regression overall depression attitude (R-DAQ)

	Unstandardized coefficients		Standardized coefficients	*t*	Sig.
	B	Std. error	Beta		
(Constant)	45.290	1.815		24.96	<0.001
Any supernatural depression cause	3.278	0.568	0.247	5.769	<0.001
Rural Practice	2.645	0.807	0.139	3.275	0.001
Divorce depression cause	−1.652	0.581	−0.121	−2.841	0.005

Dependent variable: R-DAQ score

Similar regression models were fitted for the R-DAQ subscales. Variation in the generalist sub-scale was explained by a combination of supernatural causal beliefs, psychiatric study as a major topic, psychiatry CME and rural practice (all of which exerted a significant negative influence); the overall model accounted for 16 % (R^2^ = 0.160) of the variance in this generalist perspective (F_4,525_ = 24.963, *p* < 0.001); psychiatry CME had the largest (negative) effect (β = 0.197).

For the professional confidence sub-scale, a smaller degree of variance (R^2^ = 0.043) was explained by a model (F_3,523_ = 7.825, *p* < 0.001) incorporating recent graduation (since 2012), together with having undertaken psychiatry CME, and rejecting supernatural causes of depression (each of which exerted significant positive effects). More recent graduation was the most significant factor in this model (β = 0.151).

Improved therapeutic optimism (R^2^ = 0.056), was similarly explained by a model incorporating the rejection of supernatural beliefs, together with working in an urban setting and seeing divorce as potential cause of depression (F_3,515_ = 10.107, *p* < 0.001). These were all significant predictors of therapeutic optimism, but eschewing supernatural beliefs accounted for the largest variance in this model (β = 0.172).

For these analyses the tolerance (0.923 to 0.994) and variance inflation (1.007 to 1.083) did not indicate any multicollinearity problems.

## Discussion

### Main findings

In this study of Pakistani medical practitioners based in Lahore we identified that although there are high levels of agreement about standard biopsychosocial causes for depression such as work and study stressors, poverty, and divorce, a relatively large proportion (just over a third) of doctors also maintain alternative explanatory models involving religious or supernatural forces such as the evil eye and God’s punishment. An unexpected finding was that greater exposure to psychiatry study within medical training appeared associated with an increased likelihood of holding supernatural explanatory beliefs about depression, though of course it is impossible to infer any direction of causality in this apparent relationship. Depression attitude responses measured by the R-DAQ revealed that although the participants had a generally positive view of the effectiveness of medication and talking treatments for depression and of its responsiveness to treatment, the view that depression was related to personal weaknesses and a natural part of growing old were widely held.

Through the use of the R-DAQ scale alongside items relating to depression causes, we have identified particular associations between causal beliefs and attitudes about the nature and treatability of depression. Overall it appears that for clinicians in this study, less confident, less inclusive and more pessimistic attitudes to depression are associated with holding supernatural beliefs about the cause of depression. This relationship was evident for the total R-DAQ score, and for each of the three subscales. Regression analyses controlling for other measured variables indicate that this was a robust relationship, with supernatural beliefs a significant factor for total R-DAQ and sub-scale scores, and remaining significant when controlling for other factors. For the total R-DAQ score and for the therapeutic optimism sub-scale, rejecting supernatural explanations accounted for the largest proportion of the variance in the regression models.

### Comparison with other study findings

Studies that have examined attitudes to mental illness in general and to depression in particular have identified differences related to culture and nationality: a representative general population survey (*n* = 4011) conducted in four European nations revealed substantial differences in stigmatising views about depression voiced by participants. In this telephone survey, the views that people with depression could ‘snap out of it if they wanted’ and that depression was a sign of personal weakness were agreed with by two to three times as many people in Hungary as in Ireland and Germany, and these differences between countries remained after controlling for other variables [37].

Research that has explored explanatory models of depression has similarly identified that these are strongly influenced by culture, with supernatural causal explanations much more commonly held in non-Western than Western cultures. A systematic review of relevant studies among lay people revealed magical, religious and spiritual explanations to be frequently held by people in Asian and African nations [38]. Among the Pakistani population, a recent survey of university students’ (*n* = 527) attitudes toward mental illness identified that around a quarter of respondents believed that demonic possession, the evil eye or God’s punishment were causes of mental illness, whilst nearly a third noted that black magic was a cause [28]; as in the current study, endorsement of supernatural beliefs was associated with more negative and stigmatising attitudes to mental health problems. Exploration of the beliefs of different ethnic groups living in Western countries indicates the maintenance of such explanations despite acculturative influences: a survey examining beliefs about depression causes among Bangladeshi and White people in East London (*n* = 364) revealed supernatural explanations such as black magic to be widely held among the Bangladeshi participants, irrespective of their age [39].

Similar differences related to nationality and culture are evident in studies of health professionals. A survey of medical students in the UK (*n* = 760) using on-line survey methods indicated that more stigmatizing views about psychosis were evident in Chinese and Asian students than their White British counterparts [40]; whilst a study of registered nurses in mental health settings in five European countries (*n* = 810) indicted substantial differences in perceived dangerousness and restrictive attitudes related to nation of practice [41, 42]. Fewer studies have been conducted within non-Western cultures, however, a survey medical students and doctors (*n* = 294) in three medical colleges in Lahore, Pakistan, identified that just over half of the respondents held negative attitudes towards people with schizophrenia, depression, drug and alcohol disorders [20]. For depression, a majority of respondents noted that patients had only themselves to blame (76 %) and should be able to pull themselves together (54 %): these negative attitudes to depression were much more prominent than those revealed in a UK general population survey that used the same questions [43].

Comparison of the R-DAQ depression attitude findings of this Pakistani sample of doctors with the R-DAQ validation study sample, which was comprised of mostly nurses, together with GPs and other health professionals, almost entirely based in the UK (*n* = 1193) [29], indicates significantly less positive views about depression among the Pakistani practitioners. For every item of this 22 item scale, mean scores in the Pakistani sample were more negative, and the total scale score and the scores for each of the sub-scales were substantially lower. The largest differences were for items concerning depression being due to a lack of stamina or will-power, or a natural part of old age and adolescence, or that its recognition and management are an important part of broader health care.

For the eight R-DAQ items derived from the original DAQ, comparison with the findings of studies involving general medical practitioners in the UK, Italy and France and published between 1999 and 2008 [31] and in Japan in 2009 [44] indicates that the doctors in the current study hold fairly similar views concerning the potential for therapeutic change in depression (items 18 and 21), but are markedly more likely to see this condition as a consequence of aging (item 9) and due to personal weakness (item 12). The extent of feeling comfortable addressing depressed patients’ needs, and finding this rewarding (items 1 and 19), were fairly similar between Pakistani and European clinicians (whilst none of the Japanese doctors indicated that they felt comfortable in this role).

Despite harbouring more negative and deterministic views of depression, the Pakistani doctors indicated a similar level of confidence about the efficacy of antidepressant (item 4) and psychological (item 3) therapies as doctors from the UK and European nations, with medical practitioners in Japan showing substantially less confidence in these treatments.

### Strengths and weaknesses

Rather than a probability sample of doctors representative of the medical staff in Pakistan, this study was based on a convenience sample of doctors based in six institutions within a single city; as such caution must be exercised in generalisations based on these findings as selection bias may affect the characteristics and responses of this sample. The approach to participant selection involved seeking the involvement of all available medical staff within the selected hospitals, and the high response rate (86 %) together with the modest number of incomplete questionnaires provide some assurance of the representativeness of the data obtained. Items concerning possible causes of depression were the most commonly omitted and affected between 3 and 14 % of questionnaires; comparison of the characteristics of participants who completed all items and those who omitted these items revealed no differences in age, gender, field of practice, or training.

The sample size was based on available data concerning the measures used, and the recruited sample was sufficient for all the planned analyses and provided the required level of precision.

The study used the R-DAQ, a scale which was psychometrically validated with a sample largely from the UK. This first use of this measure among Pakistani doctors indicated that, although the factor structure and many of the items were appropriate, several items fitted weakly with this population, and the internal consistency, though mostly adequate, was weaker than in the initial development study.

This study used similar survey items about depression causal factors to a previous study [28], and this, together with the use of the R-DAQ, has enabled direct comparison with other studies. Furthermore, the use of items from the original DAQ in the revised version, has allowed further (item-level) comparison of the current findings with those from a larger number of studies from different nations.

### Implications and recommendations

The finding that a substantial proportion of doctors practicing in Lahore maintain some level of belief in supernatural causes for depression, and that holding such causal beliefs is associated with more pejorative attitudes to this condition and its management, merits attention. Addressing negative and pessimistic attitudes is a key part of efforts to dispel mental health stigma and associated discriminatory behaviours, and increased understanding of the ways that these views may be linked to culture and belief will be useful in informing approaches to education for health professionals as well as the wider public. However, the complexity of this area indicates that further studies are needed to better understand the factors that inform explanatory models for depression, as well as to examine the effect of beliefs and attitudes on actual clinical behaviours. In-depth exploration of clinicians’ views provided by qualitative research would usefully complement the findings of this study, as would observational studies to examine associations between clinicians’ attitudes and their consultation styles and treatment and referral decisions.

## Conclusion

Misinformation and negative perceptions among the general public about mental health problems are common and lead to discrimination and social exclusion. These negative attitudes are not restricted to the public, and this study adds to research that has examined the views of clinicians and identified varying levels of mental health related stigma [3]. The views of general medical staff are particularly important for the accurate identification of depression because this is a common condition in primary care and general medical settings where there is substantial comorbidity with medical conditions, particularly those that are long-standing [45]. Negative attitudes are likely to influence condition recognition and the subsequent provision of appropriate advice, treatment and support, which may result in increased morbidity and mortality [46]. Critical and pejorative views held by clinicians about the nature, cause and potential outcomes of depression will contribute to internalized and treatment stigma and may deter help-seeking among the public [5].

This relatively large-scale study of Pakistani medical practitioners has provided new insights into the relationships between their explanatory models of depression and their attitudes to its assessment and management. The relationship between culture and beliefs is complex, and local representations and concepts of mental illness inevitably shape attitudes and behaviour: the medical practitioners in this study have been influenced by cultural and religious traditions as well as by their professional education and role. This research has improved our understanding of the way these factors inter-relate and may assist the development of health services and public and professional health education strategies that, whilst culturally sensitive, are effective in reducing stigma and promoting effective mental health care.
